# Investigating the relationships of structural and functional neural networks of primary visual cortex with engineered AAVs and chemogenetic-fMRI techniques

**DOI:** 10.7150/thno.109625

**Published:** 2025-03-03

**Authors:** Zhu Gui, Kunzhang Lin, Nengsong Luo, Zengpeng Han, Zhuang Liu, Ziyue Zhao, Lei Gu, Fanyong Xu, Ning Zheng, Fuqiang Xu, Xihai Li, Jie Wang

**Affiliations:** 1 Department of Radiology, Songjiang Research Institute, Shanghai Key Laboratory of Emotions and Affective Disorders, Songjiang Hospital Affiliated to Shanghai Jiao Tong University School of Medicine, Shanghai, China.; 2 State Key Laboratory of Magnetic Resonance and Atomic and Molecular Physics, Innovation Academy for Precision Measurement Science and Technology, Chinese Academy of Sciences, Wuhan 430071, China.; 3 University of Chinese Academy of Sciences, Beijing 100049, China.; 4 Shenzhen Key Laboratory of Viral Vectors for Biomedicine, Shenzhen-Hong Kong Institute of Brain Science, Shenzhen Institutes of Advanced Technology, Chinese Academy of Sciences, 518055 Shenzhen, China.; 5 Wuhan National Laboratory for Optoelectronics, Huazhong University of Science and Technology, 430074 Wuhan, China.; 6 Department of Pediatrics, Boston Children's Hospital, Harvard Medical School, Boston, MA, USA.; 7 Clinical & Technical Support, Philips Healthcare, Wuhan, China.; 8 Academy of Integrative Medicine, College of Integrative Medicine, Afffliated Third People's Hospital, Fujian University of Traditional Chinese Medicine, Fuzhou, China.

**Keywords:** neural circuits, MRI, aquaporin 1 (AQP1), AAV, whole brain, primary visual cortex (V1)

## Abstract

**Rationale:** Neural networks are crucial for brain function, but the relationship between functional and structural networks remains unclear, hindering disease understanding and treatment. This study employs AAVs, MRI reporter gene (AQP1), and chemogenetic-fMRI to explore the relationship between structural and functional connectivity in the mouse primary visual cortex (V1), offering a novel approach to study abnormal brain functions.

**Methods:** The rAAV-PHP.eB-DIO vector encoding AQP1 was used to cross the blood-brain barrier and infect the brain, enabling *in vivo* diffusion-weighted imaging (DWI-MRI) to assess the anterograde structural connectivity network of V1. Additionally, chemogenetic activation of V1 using a Cre-dependent system was performed, and the whole-brain BOLD responses were evaluated using fMRI. The integration of these techniques provided a comprehensive analysis of the relationship between functional and structural connectivity.

**Results:** This study successfully achieved the combined detection of structural and functional connectivity in the V1 of mice. The rAAV1-hSyn-Cre virus was utilized for monosynaptic anterograde tracing. Additionally, a blood-brain barrier-crossing serotype viral vector, rAAV-PHP.eB-DIO-AQP1-EGFP, was intravenously injected to effectively transduce AQP1 into the V1 region and its downstream areas. The results indicated that regions expressing AQP1 under the control of Cre recombinase, including V1, LGN, CPu, and SC, exhibited significant alterations in DWI signal intensity (SI) and apparent diffusion coefficient (ADC). DREADD-fMRI analysis revealed that chemogenetic activation of the V1 region significantly enhanced neural activity in related brain regions, accompanied by a notable increase in BOLD signals. These regions included CPu, HIP, TH, SC, and PAG.

**Conclusion:** *In vivo* decoding of neuronal activity and structural connectivity provides insights into brain structure-function interplay, which was important for understanding the cerebral function.

## Introduction

Psychiatric disorders are always related to the abnormalities in the neural circuits 1. Various neural circuits were constituted of structural and functional networks, which have gained increasing attention 2, 3. The complex neural circuits constitute the foundational framework that enables the brain to perform a wide spectrum of functions, ranging from basic to advanced. Disruptions in neural network connectivity may lead to neurological disorders, including Parkinson's disease, Alzheimer's disease, and schizophrenia 4-6. However, interpretation of the functional networks is mostly based on the neural structural connectivity, which introduces significant bias. Thus, it is important to develop a novel method to investigate the functional network based on the whole brain. Furthermore, deciphering the structural and functional connectivity of brain networks is also crucial for understanding brain function under both physiological and pathological conditions.

Currently, a range of advanced labeling and imaging techniques have been developed to investigate the neural circuitry. Notably, viral vector-based neural tracing methodologies have been extensively employed to elucidate the anatomical connectivity between brain regions. These viral tools are derived from engineered strains of naturally occurring neurotropic viruses, including herpes simplex virus (HSV), rabies virus (RV), pseudorabies virus (PRV), and vesicular stomatitis virus (VSV) 7-9. The recombinant adeno-associated virus (rAAV) vector, engineered from wild-type AAV, is the most widely used viral tool in biomedicine, with 13 unique AAV serotypes (AAV1-13) identified across various host species 10. The anterograde, retrograde, and bidirectional transport properties of AAV are intricately linked to its serotype and concentration 10-12.

Certain AAV serotypes, such as AAV1 and AAV9, have been shown to facilitate anterograde trans-synaptic transmission. High-titer AAV1-Cre can effectively infect presynaptic neurons and selectively drive Cre-dependent gene expression in postsynaptic neurons, thereby enabling the tracing and functional modulation of directly connected output neurons 13. These variants are particularly effective for the labeling of projection neurons with defined outputs. Advances in AAV capsid engineering have led to the development of variants such as AAV-PHP.B 14 and AAV-PHP.eB 15, which exhibit markedly enhanced permeability across the blood-brain barrier. Consequently, these variants are exceptionally well-suited for facilitating comprehensive neuronal transduction throughout the brain *via* systemic intravenous injection.

Traditional neurotropic viral tracing methods primarily employ optical imaging to investigate brain structural networks, necessitating post-mortem slices or transparentized tissue for visualization of viral-labeled circuitry. In contrast, magnetic resonance imaging (MRI) is a non-invasive technique that generates cross-sectional images of internal structures using non-ionizing electromagnetic radiation 16. MRI enables *in vivo* imaging of animal brains, with T1 and T2 weighted imaging providing detailed anatomical information and superior contrast between gray matter (GM) and white matter (WM) 17. However, diffusion tensor imaging (DTI) is limited in its ability to specifically trace distinct neural circuits, despite effectively mapping structural connectivity 18.

Protein-based contrast agents can be gene-encoded within cells to visualize the functions of specific cells and molecules *in vivo* 19-21. Ferritin is a metal protein that sequesters paramagnetic metal ions and is employed as a T1 or T2 weighted MRI contrast agent 22, 23. In previous studies, the ferritin gene was incorporated into vesicular stomatitis virus (VSV), demonstrating a multi-level neural network connected to the sensory cortex 24. The retrograde transport AAV vector encoding ferritin enables the dissection of neural networks *in vivo* and allows for long-term monitoring of viral infections 25. Relative to conventional metal-binding protein reporter genes, the non-metal MRI reporter gene aquaporin 1 (AQP1) exhibits reduced cytotoxicity and enhanced sensitivity 19. Prior research has employed DWI-MRI to detect the* in vivo* single-level network retrogradely connected to the caudate putamen (CPu), labeled with AQP1 encoded by rAAV2-retro 26. However, there is still a notable deficiency in MRI visualization of the anterograde downstream single-level network, which is very important for the investigation of the functional network of a target brain region.

On the other side, a rapidly expanding approach for understanding brain functional networks involves non-invasive neural imaging using rsfMRI (resting-state functional magnetic resonance imaging) to map large-scale patterns of spontaneous activity 27, relying on the variations of BOLD signals (Blood Oxygen Level Dependent). The combination of BOLD signals and Local Field Potential (LFP) recordings has been used to study the response of different olfactory bulb layers to odor stimulation 28. Another study simultaneously recorded whole-brain rsfMRI and LFP from two different brain regions of rats, analyzing the spatiotemporal relationship between the two types of signals to explain the underlying neural activity based on resting-state networks (RSNs) 29. Furthermore, some studies have employed chemogenetics/optogenetics 30, in conjunction with fMRI, to investigate the relationship between local neuronal activity and global network activity 31-33. For example, DREADD (Designer Receptors Exclusively Activated by Designer Drugs)-rsfMRI was used to investigate the role of dorsolateral anterior cingulate cortex (dACC) on the whole brain Default Mode Network (DMN) activity and connectivity 34. Acute inhibition of neural activity in a central region (*i.e.* dACC) affected the topological structure of the whole-brain network, whereas no such changes were observed in a non-hub region (*i.e.* V1) 35. However, the structural basis of the associated functional networks in the whole brain remains unclear.

In this study, we utilized rAAV-PHP.eB-DIO to encode AQP1 for crossing the blood-brain barrier and infecting the brain. We administered a mixture of the anterograde single AAV vector (rAAV1-Cre) and rAAV9-EF1α-DIO-hM3D (Gq) into the V1 of mice. Subsequently, we utilized DWI-MRI to evaluate the anterograde single-level structural connectivity network of V1 *in vivo* and employed chemogenetics to activate the V1 region, examining the resulting whole-brain BOLD response and its associated functional network. By integrating the structural and functional networks of specific brain regions, we gained a deeper understanding of the relationship between neuronal activity and structural connectivity in the brain.

## Materials and Methods

### Animals

All surgical and experimental procedures were approved by the Animal Care and Use Committee of the Institute of Precision Measurement Science and Technology Innovation, Chinese Academy of Sciences (APM22021A). Adult male C57BL/6 mice (6 weeks old) were purchased from Hunan SJA Laboratory Animal Company. All animals were housed in a quiet environment with a constant 12-hour/12-hour light-dark cycle and were randomly assigned to experimental groups. Food and water were provided *ad libitum*.

### Virus injection

For this study, the chemogenetically activating virus used was rAAV9-EF1α-DIO-hM3D (Gq)-mCherry-WPRE-pA (Brain Case, Shenzhen, P.R. China), with a titer of 2.0 × 10^12^ vg/mL. The virus used for anterograde multi-synaptic connection tracing was VSV, with a titer of 6.4 × 10^9^ PFU/mL. The anterograde transsynaptic viral tracer was rAAV1-hSyn-Cre, with a titer of 1.0 × 10^13^ vg/mL. For intravenous injections to enhance MRI contrast, the virus used was rAAV-PHP.eB-DIO-AQP1-EGFP, with a titer of 2.0 × 10^12^ vg/mL.

**Figure 1:**
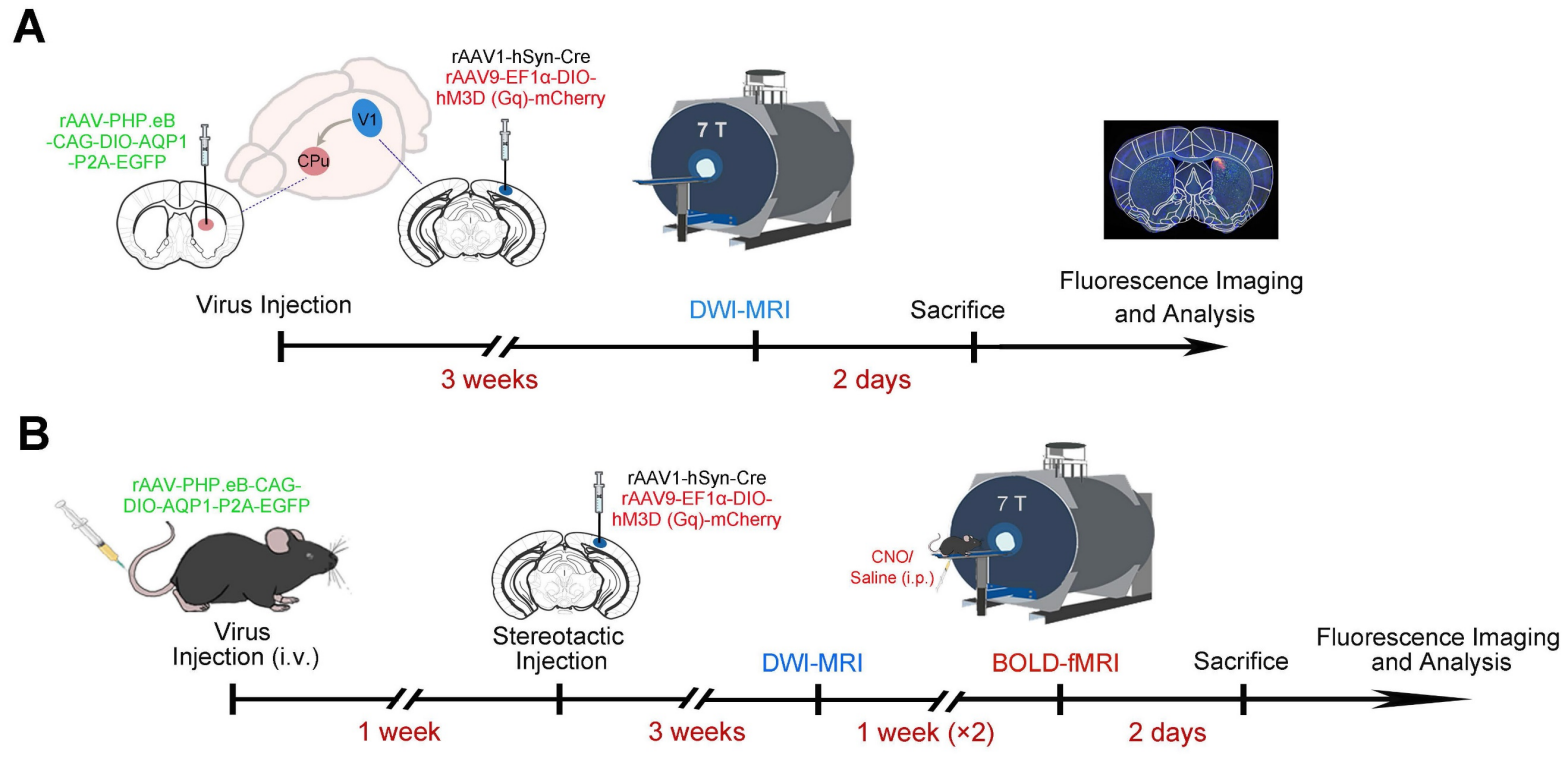
** Experimental procedure for viral injection and MRI imaging of V1 and downstream CPu neurons.** (A) A combination of Cre-dependent DREADD receptor rAAV-DIO-hM3D (Gq)-mCherry and Cre virus was injected into V1 neurons of C57BL/6 mice, while Cre-dependent rAAV-PHP.eB-DIO-AQP1-EGFP was injected into the CPu region. The experimental timeline includes viral injections, MRI scanning, and immunofluorescence imaging; (B) A combination of Cre-dependent DREADD receptor rAAV-DIO-hM3D (Gq)-mCherry and Cre virus was injected into V1 neurons of C57BL/6 mice, and Cre-dependent rAAV-PHP.eB-DIO-AQP1-EGFP was delivered systemically via tail vein injection. The timeline includes the sequence of viral injections, structural and functional network MRI analysis, and immunofluorescence imaging.

Eight-week-old male mice were anesthetized with sodium pentobarbital (50 mg/kg, intraperitoneal injection) and positioned securely in a stereotaxic frame (RWD, China). After exposing the skull, a small hole was drilled to accommodate a glass micropipette (World Precision Instruments, USA). Using coordinates derived from the mouse brain atlas (Paxinos and Franklin), 250 nL of rAAV (5 × 10^12^ vg/mL rAAV-PHP.eB-DIO-AQP1-EGFP or rAAV-PHP.eB-DIO-EGFP) was stereotactically delivered into the target region, the CPu. Additionally, 200 nL of a viral mixture (2:1 ratio) comprising rAAV1-hSyn-Cre and rAAV9-EF1α-DIO-hM3D (Gq)-mCherry-WPRE-pA, with a total titer of 5 × 10^12^ vg/mL, was injected into the V1. The stereotaxic coordinates for CPu and V1 were as follows: [CPu: A-P, 0.50 mm; M-L, -2.0 mm; D-V, -3.3 mm; V1: A-P, -3.51 mm; M-L, -2.45 mm; D-V, -0.53 mm] (**Figure 1A**). The infusion rate was maintained at 20 nL/min. Following the injection, the micropipette remained in place for an additional 20 minutes before being slowly withdrawn to minimize tissue disruption. Post-surgery, the head wound was carefully treated with lidocaine lincomycin gel (Xinya, China). For the tail vein injections, the viruses rAAV-PHP.eB-DIO-AQP1-EGFP and rAAV-PHP.eB-DIO-EGFP were diluted with PBS to the desired concentration (1.0 × 10^12^ vg/mL), and then the mixture was injected to the animal through the tail vein (**Figure 1B**).

### MRI experiment

All MRI scans were performed using a 7.0 T BioSpec horizontal-bore system (Bruker, Germany), utilizing a 20 mm diameter surface coil in combination with a birdcage transmit coil to achieve the optimal signal transmission and reception (**Figure 1**).

For the DWI-MRI experiment, mice underwent *in vivo* MRI scanning three weeks after viral injection. Anesthesia was induced with 3.5-4.0% isoflurane (RWD, Shenzhen, China) and maintained at 1.0-1.5% during the experiment. The respiration rate was monitored and kept at approximately 60 breaths per minute, while body temperature was stabilized at around 36.5 °C using a warm water pad. Scanning was performed using the 20 mm surface coil and the birdcage transmit coil for imaging the mouse brain. The DWI sequence used was a stimulated echo-based spin-echo sequence, with parameters as follows: TR = 4000 ms, TE = 24 ms, δ = 7 ms, Δ = 100 ms, b value = 1000 s·mm^-2^, FOV = 20 × 20 mm^2^, matrix size = 100 × 100, slice thickness = 0.7 mm, and slice number = 16. The total scanning time was 1 hour and 20 minutes.

For functional imaging at different time points, mice were scanned four weeks after viral injection. Anesthesia was initially induced with 3.0-5.0% isoflurane, followed by an intraperitoneal injection of 7.5 μg/mL dexmedetomidine hydrochloride solution (0.83 mL/kg). Dexmedetomidine hydrochloride served as a sedative, helping to maintain the animals in a calm state during the MRI experiment and ensuring the stability of the experimental process. The mice were then secured on the MRI animal bed, and the injection needle was connected to a catheter embedded in the mouse's abdomen for the infusion of clozapine-N-oxide (CNO) or saline during the MRI scan. The needle connected to the catheter was placed subcutaneously on the mouse's back, and dexmedetomidine hydrochloride was infused *via* a microinjection pump at a rate of 8.3 μL/min/kg. Throughout the scanning process, a circulating warm water bath was continuously used to maintain the animal's body temperature at approximately 37 °C, while a respiratory monitoring system recorded the respiratory rate.

The sequence used for BOLD fMRI was a multi-slice FLASH sequence with the following parameters: TR = 500 ms, TE = 12.5 ms, flip angle (FA) = 30°, number of averages (NA) = 2, repetition number = 50, FOV = 20 × 20 mm², matrix size = 96 × 96, and slice thickness = 0.5 mm. The total scan time was 76 minutes and 40 seconds. The sequence for T2-weighted structural images was a RARE sequence with parameters: The T2-weighted structural images were acquired using a RARE sequence, with parameters as follows: TR = 2850 ms, TEeff = 36 ms, NA = 4, FOV = 20 × 20 mm², matrix size = 256 × 256, slice thickness = 0.5 mm, and total scanning time = 6 minutes and 4 seconds.

### Slice preparation and fluorescence imaging

Mice were deeply anesthetized with an overdose of pentobarbital sodium (70 mg/kg, intraperitoneal injection) and subsequently underwent transcardial perfusion with 0.1 M phosphate-buffered saline (PBS, pH 7.4), followed by perfusion with a 4% paraformaldehyde (PFA) solution. The brain tissue was carefully extracted for post-fixation and dehydrated in 30% (weight/volume) sucrose for 24 hours. The brain was sectioned into 40-micron coronal slices using a cryostat microtome (Thermo Fisher Scientific, CRYOSTAR NX50) and stored at -20 °C. For immunohistochemical staining of AQP1 protein, brain slices were first washed three times with PBS and then blocked with a blocking solution (10% normal serum, 0.3% v/v Triton X-100 in PBS) at 37 °C for 1 hour. Subsequently, the slices were incubated with a rabbit anti-AQP1 antibody (ab219055, Abcam) overnight at 4 °C. After three washes, the slices were incubated with the secondary antibody (goat anti-rabbit CY3, diluted 1:1000, Jackson) at room temperature for 2 hours. Following three more washes, the slices were stained with DAPI (1:5000, Invitrogen), mounted with 75% glycerol (Sinopharm) in PBS solution, and sealed with nail polish. Finally, the brain slices were imaged using the Olympus VS120 virtual microscopy slide scanning system (Olympus).

### Data analysis

The original MRI data were converted to nifti format (hdr/img files) using the Bruker2Analyze tool. Subsequently, brain regions were manually delineated using ITK-SNAP software (http://www.itksnap.org). The apparent diffusion coefficient (ADC) maps were calculated in MATLAB by fitting the logarithmic decay of MRI signal intensity against the b-value. A brain template was generated using diffusion-weighted images without diffusion gradients (b0 images) and the Advanced Normalization Tools (ANTS, http://stnava.github.io/ANTs). The antsRegistrationSyN.sh script from ANTs was used to normalize the b0 images to a custom template. Each mouse's 3D volumetric image was aligned to this template through a series of rigid, affine, and nonlinear transformations to achieve inter-subject alignment. The affine and nonlinear transformation parameters were then applied to the ADC maps. The aligned ADC maps from the two groups were compared using the 3dttest++ function in AFNI (https://afni.nimh.nih.gov/). The resulting t-statistic maps were visualized using the AFNI GUI, with a significance threshold set at *p* < 0.05, and overlaid onto the custom brain template. Regions of interest (ROIs) showing significant signal were extracted, and the mean ADC values for each ROI were calculated and compared between groups using a two-tailed Student's t-test. Data were presented as mean ± standard error of the mean (Ave ± SEM). Violin plots and comparison matrices were generated to illustrate group differences, with different colors representing FDR-corrected p-values to indicate the statistical significance of inter-group differences.

Brain extraction from the T2-weighted structural images was performed manually using ITK-SNAP. The extracted brain mask was subsequently applied to the BOLD fMRI images, and the T2 mask was resampled to the EPI image space to mask the EPI images. Linear registration between the EPI images and T2 structural images was conducted using the antsRegistrationSyN.sh script from the ANTs software package. Motion correction of the EPI images was performed using the antsMotionCorr function in ANTs. N4 bias field correction and denoising were applied to the T2 structural images. The motion-corrected EPI images were registered to the T2 images using rigid body registration, and the T2 images were further registered to the standard TMBTA mouse brain template via affine and nonlinear transformations. Finally, the EPI images were standardized to the template space using the combined transformation matrices (affine and nonlinear). Spatial smoothing was applied to the standardized EPI images using a Gaussian kernel with a full width at half maximum (FWHM) of twice the voxel size. Following preprocessing, voxel-wise area under the curve (AUC) analyses for activation during the stimulation phase (volumes 25-50) were conducted using the 3dttest++ function in AFNI, comparing group differences. The resulting t-statistic maps were overlaid onto the standard template. Activation regions were segmented based on the TMBTA template, resulting in 48 regions of interest (ROIs), from which the average time series for each ROI was extracted. For both the CNO stimulation group and the Saline control group, time series were extracted and normalized to the baseline (defined as the average of the first 10 time points, equivalent to 15 minutes). The effect size was calculated using Cohen's D (representing the difference between the CNO and Saline groups), and Cohen's D maps were generated to illustrate these effects. Changes in BOLD signal intensity were represented as percentage changes relative to the baseline (average intensity of scans 1-10).

## Results

### Downstream neural circuits of V1 region

**Figure 2:**
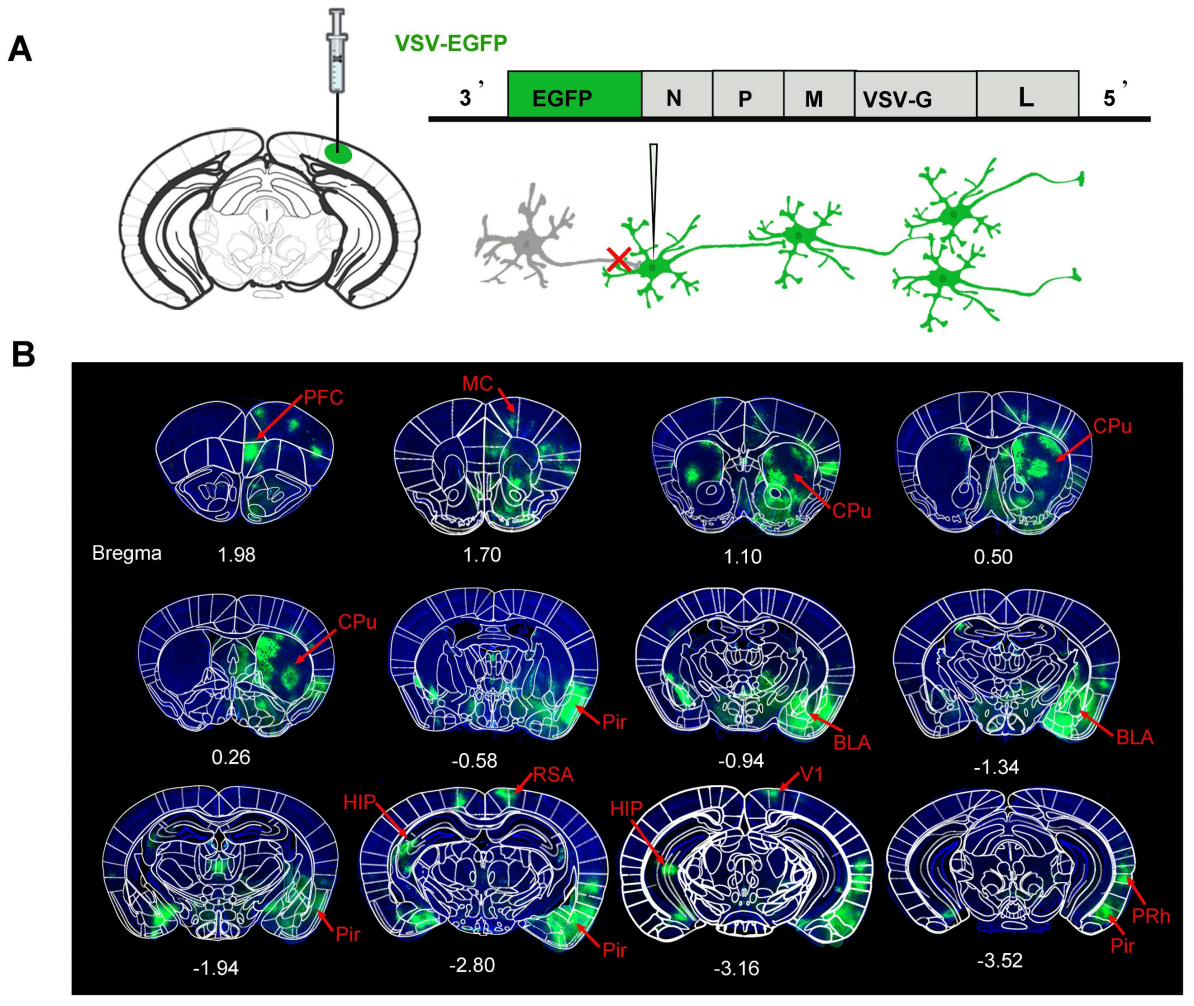
** Fluorescence images of representative slices from the whole brain after VSV-EGFP injection in the right V1 region.** (A) Diagram of the VSV-EGFP viral genome, encoding EGFP for anterograde labeling across multi-level neuronal connections; (B) EGFP expression was observed in the V1 region and its downstream areas, labeled in green to highlight the output network of neurons in the V1 region. The network includes the CPu, HIP, PFC, SC, MC, Pir, RSA, BLA and PRh. Nuclei were stained blue with DAPI.

To investigate the relationship of the structure and function networks, it is important to verify the downstream neural networks of a target brain region at first. Here, the region of V1 was selected as the target region. To investigate its downstream neural networks, we administered the neurotropic viral vector VSV-EGFP, which enables the anterograde labeling across multiple neuronal tiers, into the right V1 region of C57BL/6 mice (**Figure 2A**). This injection resulted in extensive labeling several regions direct or indirect connected with V1. The mapping results of the mouse brain indicated that the output networks of neurons for the V1 region includes CPu, bilateral hippocampus (HIP), prefrontal cortex (PFC), superior colliculus (SC), medial cortex (MC), piriform cortex (Pir), basolateral amygdala (BLA), retrosplenial agranular cortex (RSA), and perirhinal cortex (PRh), *etc*. which was collected in **Figure 2B**.

### Changes of DWI contrast in CPu region induced by AQP1 expression

To investigate the function network of a target brain, a virus tool with anterograde trans-monosynaptic tracing property was necessary. Here, rAAV1-hSyn-Cre was selected, due to its application in the area of anterograde trans-monosynaptic tracing. To investigate the trans-synaptic efficiency of rAAV1-hSyn-Cre, the V1 region and its downstream CPu region were selected as the target areas (**Figure 1A**). A mixture of rAAV9-EF1α-DIO-hM3D (Gq)-mCherry, enabling localized expression with the expression of mCherry, and the neuron-specific rAAV1-hSyn-Cre was delivered to the V1 region. Concurrently, rAAV-PHP.eB-DIO-AQP1-EGFP and the control virus rAAV-PHP.eB-DIO-EGFP were separately injected into the right CPu region of two groups of mice. The mCherry expression was confined to the V1 region, marking the injection site. Three weeks post-infection, the *in vivo* imaging of the mouse brain was conducted using an MRI scanner. After the MRI scans were completed, the mice were perfused to obtain brain sections for further analysis (**Figure 1A**, *n* = 5).

**Figure 3:**
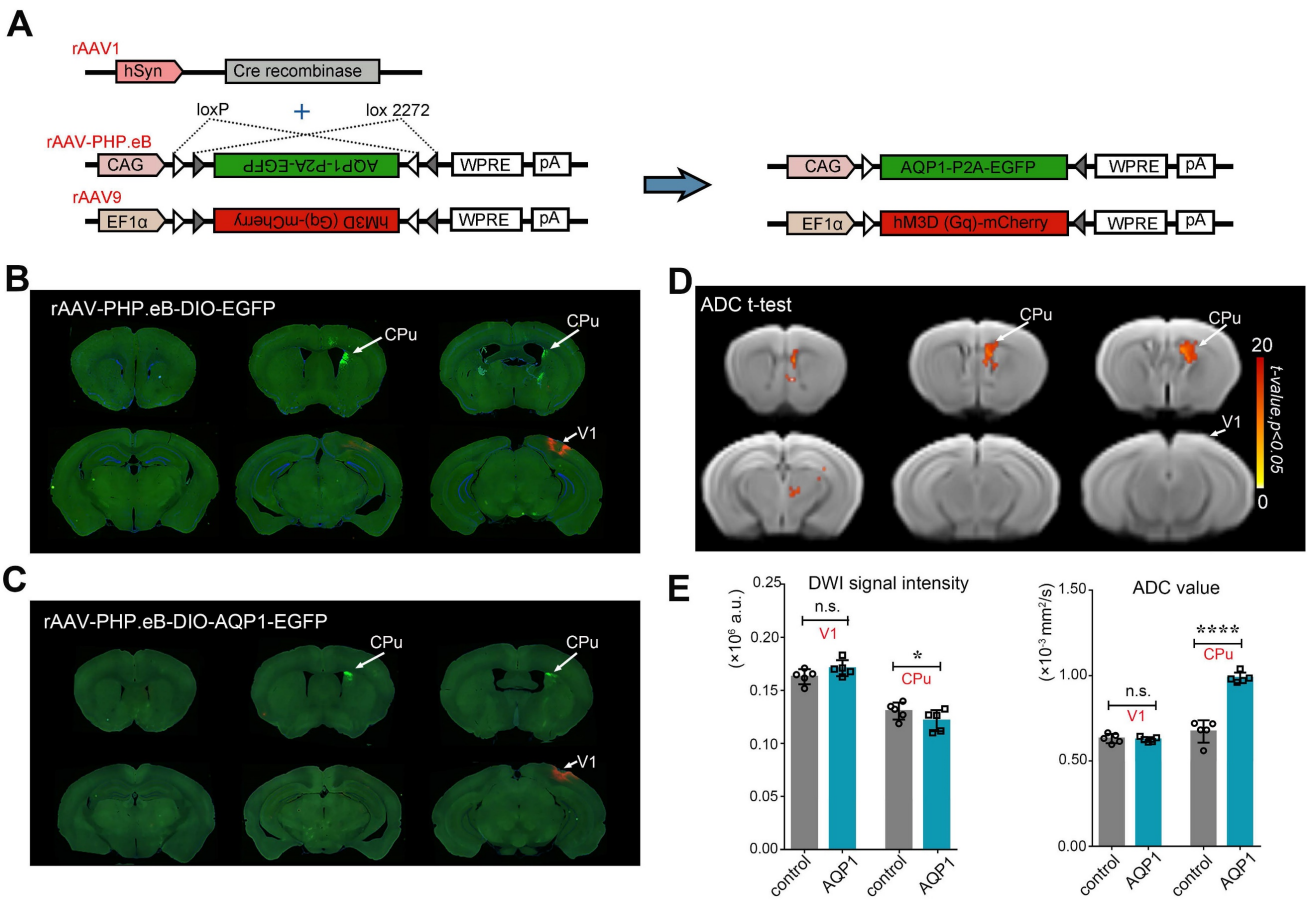
** DWI-MRI and fluorescence imaging of mice injected with rAAV-PHP.eB-DIO-AQP1-EGFP or DIO-EGFP in the CPu region.** (A) Schematic diagram of the rAAV-PHP.eB viral genome, encoding the Cre-dependent AQP1-EGFP gene. Gray and white triangles represent two distinct *loxP* sites that enable AQP1-EGFP expression in the presence of Cre recombinase. The expression of AQP1-EGFP and hM3D (Gq)-mCherry occurs exclusively in Cre-positive cells; (B-C) EGFP fluorescence images of mouse brains in the rAAV-PHP.eB-DIO-EGFP group (B) and the rAAV-PHP.eB-DIO-AQP1-EGFP group (C); (D) The ADC values of mouse brains injected with rAAV-PHP.eB-DIO-AQP1-EGFP were compared to those injected with rAAV-PHP.eB-DIO-EGFP using voxel-by-voxel t-tests. The color bar indicates the t-values obtained from the Student's t-test, where brighter shades represent more significant differences. (E) Quantitative analysis of DWI signals and ADC values extracted from ROIs, including the V1 and CPu regions, for both the AQP1-EGFP and EGFP groups. *n* = 5, * p < 0.05; ** *p* < 0.01; *** p < 0.001; **** p < 0.0001.

The Cre virus infected neurons in the V1 region and propagated along their axons, specifically driving Cre-dependent expression of AQP1 in the neurons of the CPu region, one of its monosynaptic downstream targets (**Figure 3A**). DWI signals from the brains of mice injected with rAAV-PHP.eB-DIO-EGFP (**Figure 3B**) and rAAV-PHP.eB-DIO-AQP1-EGFP (**Figure 3C**) were acquired, and ADC maps were generated. Voxel-wise comparisons of ADC values between groups were performed, with significant t-values visualized on a custom template using a color scale (**Figure 3D**). To quantify DWI contrast changes in the V1 region and the CPu region expressing AQP1, DWI signal intensities and mean ADC values of the two areas were calculated and compared. Results showed no significant differences in DWI signal or ADC values in the V1 region without AQP1-EGFP expression compared to the control group. However, the CPu region expressing AQP1 demonstrated significantly lower DWI signal values and higher ADC values relative to the control group (**Figure 3E**).

**Figure 4:**
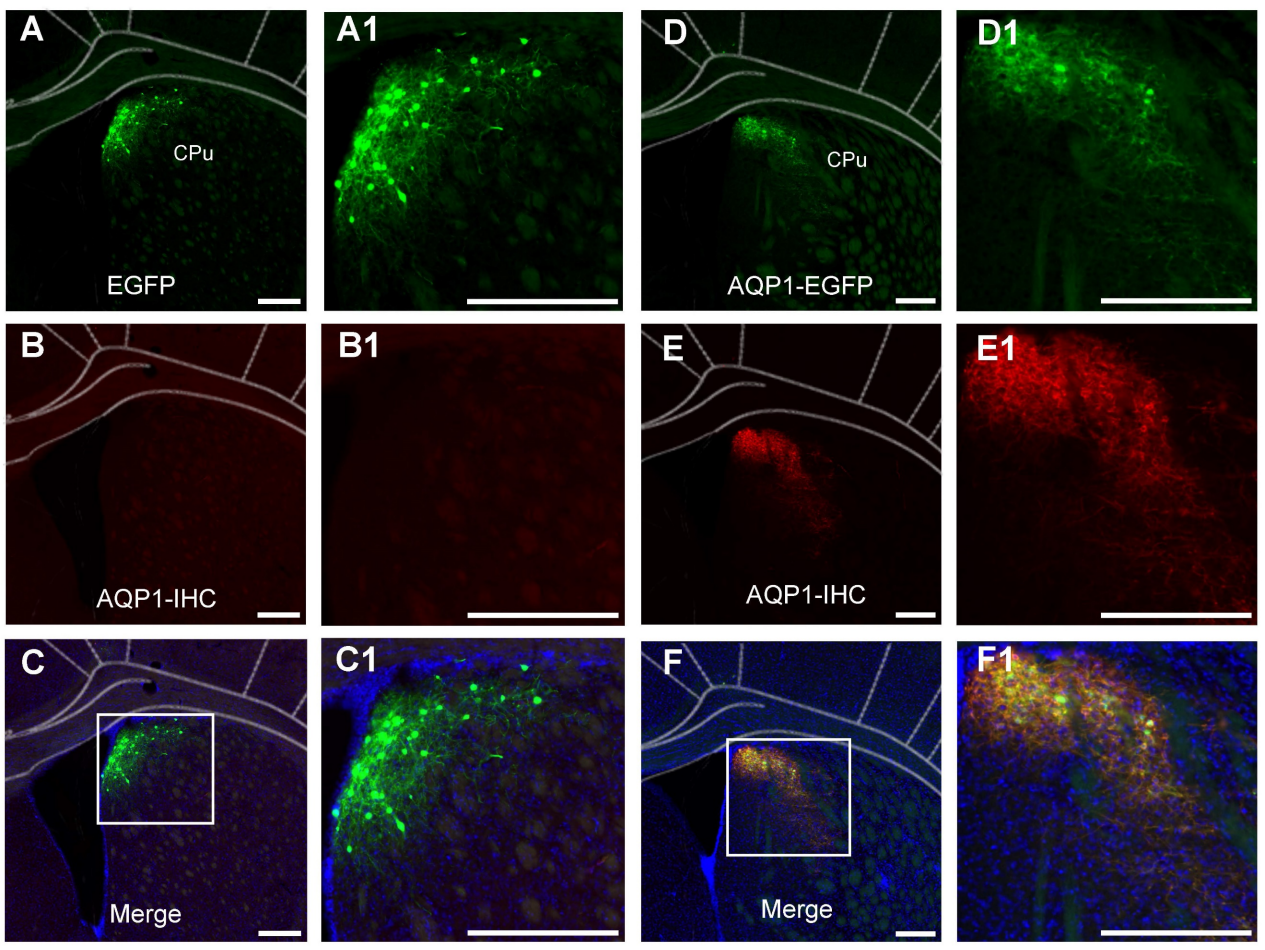
** Immunofluorescent staining of neurons in the CPu region infected with rAAV-PHP.eB-DIO-AQP1-EGFP or rAAV-PHP.eB-DIO-EGFP.** (A-C) In the EGFP group, representative coronal brain sections show virus-labeled neurons in the CPu region (green, A), AQP1 immunofluorescent staining (red, B), and co-localized neurons (yellow, C). No red fluorescence was observed in the control group. Panels A1, B1, and C1 are higher-magnification images of the boxed regions in panels A, B, and C, respectively; (D-F) In the AQP1-EGFP group, the CPu region shows virus-labeled neurons (green, D), AQP1 immunofluorescent staining (red, E), and the merged results of co-localized labeling (yellow, F). The results demonstrate that AQP1 protein and EGFP protein are expressed in the same neurons in the CPu region of the AQP1-EGFP group. Panels D1, E1, and F1 are higher-magnification images of the boxed regions in panels D, E, and F, respectively. *Nuclei were stained blue with DAPI*.* Scale bar*: 200 μm.

To verify that the regions displaying green fluorescence express the AQP1 protein, immunohistochemistry was performed to localize AQP1 protein in brain sections (**Figure 4**). The co-localization of AQP1 and EGFP indicated that AQP1 was specifically expressed in the CPu region, which was infected with rAAV-PHP.eB-DIO-AQP1-EGFP (**Figure 4A-F**).

### Anterograde primary downstream areas of V1 induces changes in DWI contrast

By conducting *in vivo* tracing of the downstream CPu region of V1, the trans-synaptic transmission capability of rAAV1-hSyn-Cre and the potential of AQP1 for MRI imaging have been demonstrated. To investigate the anterograde monosynaptic neural connectivity across the entire brain in live animals, a blood-brain barrier-crossing serotype rAAV-PHP.eB-DIO-AQP1-EGFP viral vector was administered *via* tail vein injection in C57BL/6 mice. After one week of viral infection in the brain, stereotactic injections were performed in the V1 region using a retrograde monosynaptic AAV vector (rAAV1-Cre) to trace its downstream primary targets. Following an additional three weeks of viral expression, DWI was utilized to visualize the brains of the live mice (**Figure 1B**, *n* = 6).

**Figure 5:**
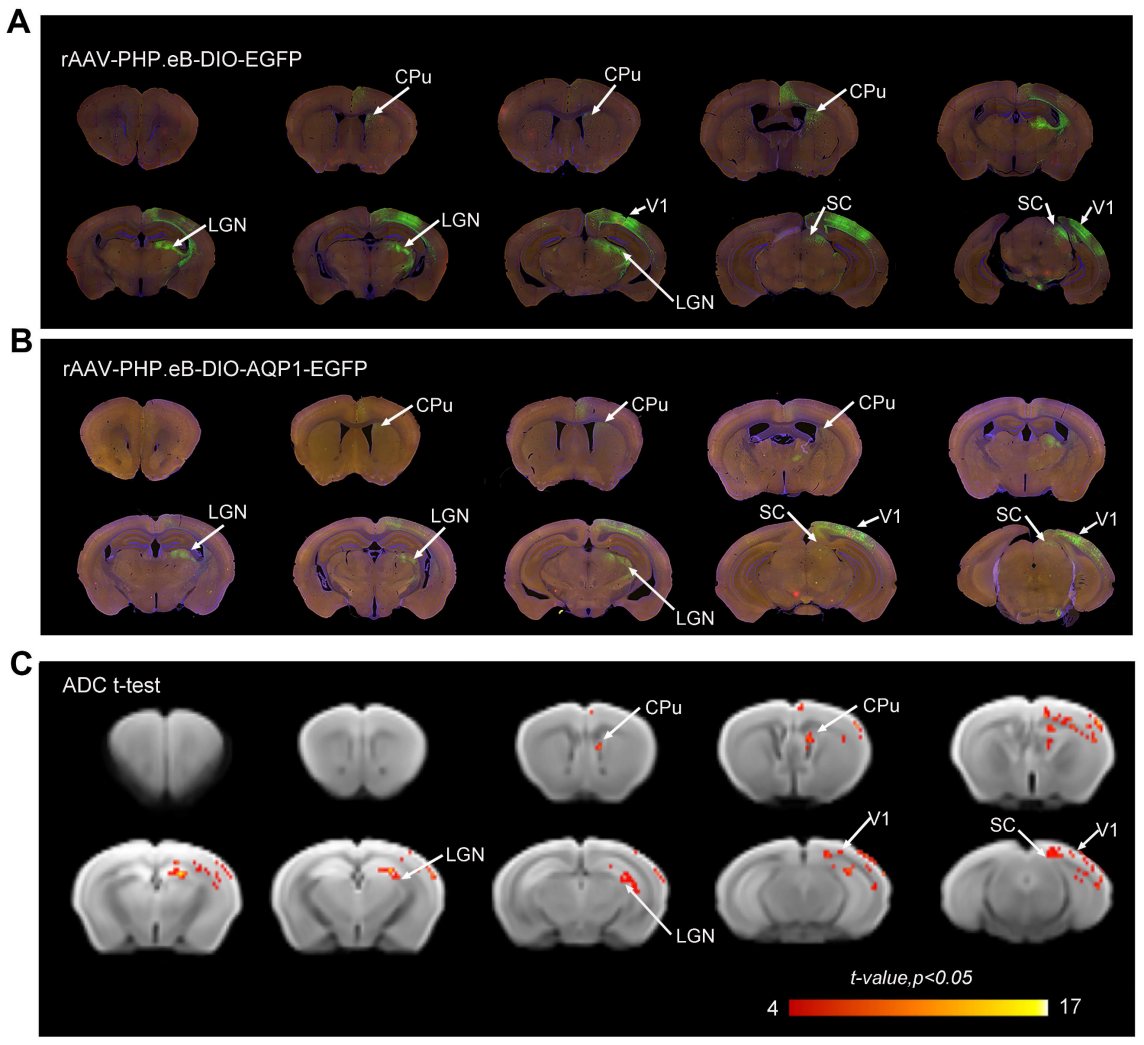
** DWI-MRI and fluorescence imaging of the anterograde monosynaptic network in mouse brains injected via the tail vein with rAAV-PHP.eB encoding DIO-AQP1-EGFP or DIO-EGFP.** (A-B) Representative whole-brain coronal sections showing EGFP fluorescence in the rAAV-PHP.eB-DIO-EGFP group (A) and the rAAV-PHP.eB-DIO-AQP1-EGFP group (B). Fluorescent signals were observed in several brain regions, including the right V1, CPu, LGN, and SC; (C) ADC values of mouse brains injected with rAAV-PHP.eB-DIO-AQP1-EGFP were compared to those injected with rAAV-PHP.eB-DIO-EGFP using voxel-wise t-test. The color bar represents the t-values from the Student's t-test, with brighter shades indicating greater differences. *n* = 6,* p* < 0.05.

The patterns of EGFP signal in brain sections from both groups were similar, indicating that the AQP1-EGFP carried by the rAAV-PHP.eB vector and the control EGFP effectively infected the similar brain regions, including V1, SC, CPu, and Lateral Geniculate Nucleus (LGN) (**Figure 5A, B**), consistent with previously reported downstream monosynaptic regions from V1 13. DWI scans were obtained for both groups of mice, and ADC maps were generated. Voxel-wise comparisons of ADC values between the groups were performed, and significant t-values were visualized on a custom template using a color-coded scale (**Figure 5C**). The results showed that regions with significant differences in ADC values largely corresponded to areas exhibiting green fluorescent signals.

### Analysis of the impact of AQP1 expression on ADC changes in brain regions

**Figure 6:**
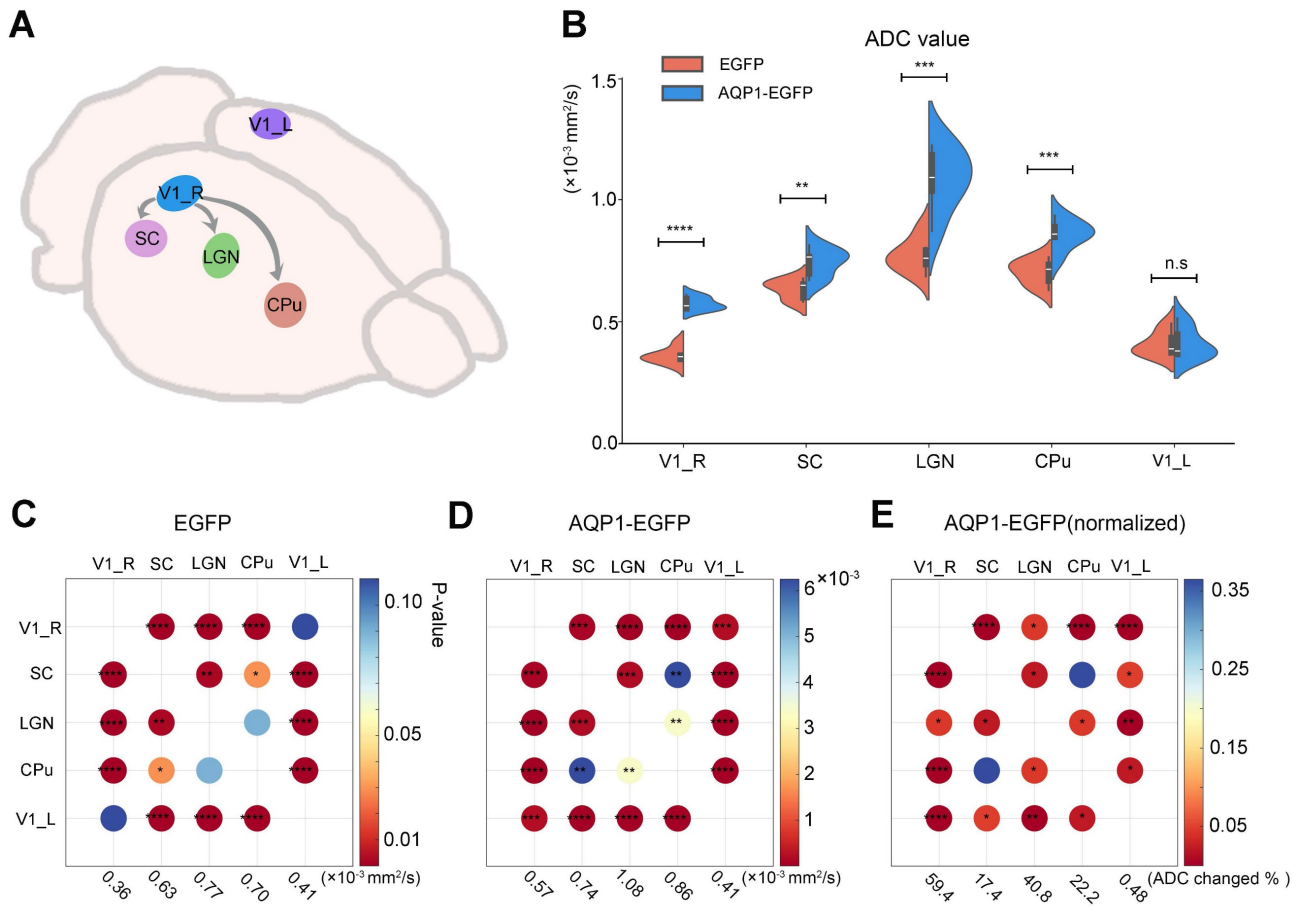
** Comparison of ADC values in AQP1 transduction areas after rAAV- PHP.eB-DIO-AQP1-EGFP or rAAV-PHP.eB-DIO-EGFP Injection into the V1 region.** (A) Regions of interest (ROIs) in the mouse brain, including the AQP1 transduction areas and the non-transduced left V1; (B) ADC values of each region were analyzed by t-test, with the left V1 (V1_L) serving as a negative control; (C) Comparison matrix of increased BOLD signals in the EGFP group across the right V1, SC, LGN, CPu, and left V1. The increases in BOLD signals within activated brain regions were calculated and compared pairwise. The color of each dot indicates the *p*-value; (D) Comparison matrix of increased BOLD signals in the AQP1-EGFP group across the right V1, SC, LGN, CPu, and left V1. The increases in BOLD signals within activated brain regions were calculated and compared pairwise. The color of each dot indicates the *p*-value; (E) Comparison matrix of normalized BOLD signal increases (normalized to the EGFP group) in the AQP1-EGFP group across the right V1, SC, LGN, CPu, and left V1. The increases in BOLD signals within activated brain regions were calculated and compared pairwise. The color of each dot indicates the *p*-value. Error bars represent the mean ADC values ± standard error, with FDR correction*.* * *p* < 0.05; ** p < 0.01; *** p < 0.001; **** p < 0.0001.

To assess the alterations in DWI contrast within AQP1-expressing regions, brain regions exhibiting significant inter-group differences in ADC values were extracted (**Figure 6A**), and the mean ADC values of these regions were calculated and compared. Compared to the regions infected with rAAV-PHP.eB-DIO-EGFP, the brain regions infected with rAAV-PHP.eB-DIO-AQP1-EGFP, including those transduced with AQP1 (SC, CPu, and LGN), exhibited significantly higher ADC values. The uninfected left V1 region was selected as a negative control, as no green fluorescence signal was observed in this region and no significant changes in DWI contrast were detected. For the rAAV-PHP.eB-DIO-AQP1-EGFP group, the ADC of AQP1-expressing brain regions was significantly increased (*p < 0.0001*), while the ADC of the left V1 region showed no significant difference compared to the control group (**Figure 6B**). These results indicate that *in vivo* DWI successfully traced the downstream neural connections of the V1 region after rAAV1-Cre was combined with the rAAV-PHP.eB-DIO-AQP1-EGFP vector.

To compare the degree of AQP1-mediated diffusion enhancement among different brain regions, intra-group paired comparisons of ADC changes were conducted for the control group and the AQP1-transduced group. In the rAAV-PHP.eB-DIO-EGFP control group, no significant differences in ADC were observed between the left and right V1 regions (**Figure 6C**). However, in the rAAV-PHP.eB-DIO-AQP1-EGFP group, a significant difference in ADC was found between the right V1 region and the left V1 negative control region (**Figure 6D**). To eliminate potential discrepancies due to baseline ADC signals across different brain regions, normalization of the ADC values in the AQP1-transduced group was performed using the control group as a reference (**Figure 6E**). The results showed a highly significant difference in ADC between V1 and the two regions, SC and CPu, while the difference between SC and CPu regions was not significant. This lack of difference may be attributed to variations in the connectivity strength between brain regions.

### Whole-brain BOLD responses induced by activation of neurons in the V1 region

**Figure 7:**
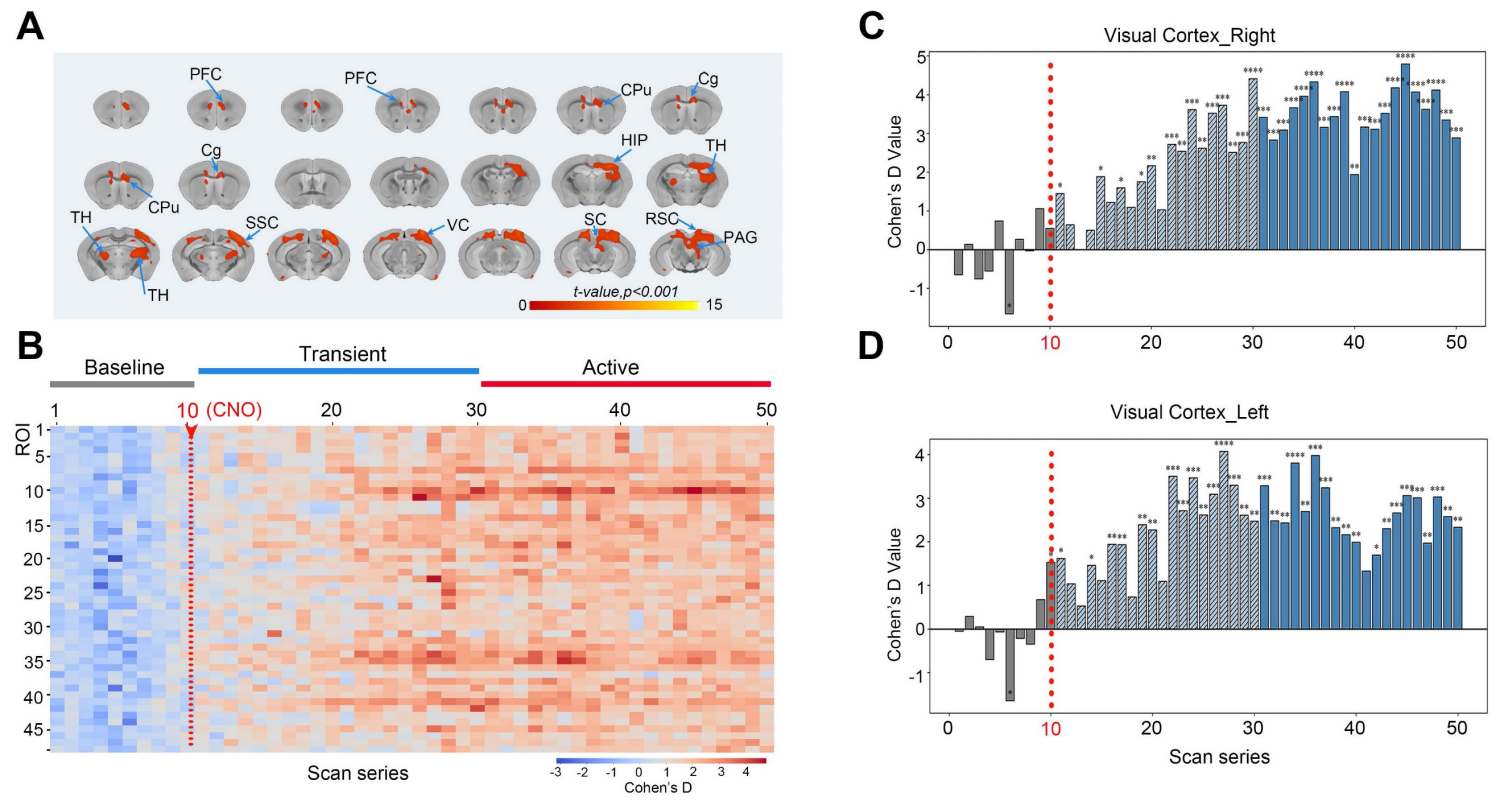
** Responses of the BOLD signals following the chemogenetic activation of neurons in the V1 region with the injection of CNO.** (A) The neural activity map shows brain regions with significant BOLD signals following DREADD-mediated chemogenetic activation of the V1 region. These regions include the right VC, contralateral VC, SC, RSC, PAG, SSC, TH, CPu, Cg, and PFC; (B) Heatmap of effect sizes (Cohen's D) for 48 regions of interest (ROIs), comparing the CNO-injected group with the saline-injected group. Blue represents the baseline period (time points 1-10, minimal differences), transitioning to red during the transition period (time points 11-30), indicating increased neural activity after CNO binding to hM3D neurons. Sustained activity enhancement (red) was observed in the subsequent time points; (C-D) Effect size analysis (Cohen's d values) of the ipsilateral visual cortex (VC_R, C) and contralateral visual cortex (VC_L, D).* Note: n* = 6, repetition number = 50, one scan = 1 min 32 s; * p < 0.05; ** *p* < 0.01; *** p < 0.001; **** p < 0.0001.

BOLD-fMRI was employed to investigate the whole-brain functional network imaging following chemogenetic activation of neurons in the V1. After conducting 10 baseline fMRI scans, CNO was administered *via* intraperitoneal injection, followed by the acquisition of 40 post-injection image volumes. The analysis focused on the whole-brain BOLD responses following CNO or saline injections, with voxel-wise comparisons performed. The induced neural activity map from DREADD activation is presented in **Figure 7A**. The results of the 3dttest++ comparisons before and after activation indicated that the activated brain regions included not only the virus injection site but also the contralateral VC, SC, retrosplenial cortex (RSC), periaqueductal gray (PAG), secondary somatosensory cortex (SSC), thalamus (TH), CPu, cingulate gyrus (Cg), and PFC.

To eliminate the potential impact of intraperitoneal injection stimulation on brain activity during scanning, 48 brain regions with significant differences before and after activation were extracted (brain region numbering is shown in **Table S1**). The effect sizes for the regions of interest (ROIs) were calculated using the intergroup mean standard deviation (Cohen's D, CNO vs. saline). During the initial 10 time points, corresponding to 15 minutes (baseline), the average Cohen's D change ranged from -1 to +1, displayed as blue in the heatmap, indicating no significant signal differences. However, immediately following CNO injection, from the 11th to the 30th time point (transition period), the effect size began to increase, transitioning from blue to red in the heatmap, indicating that CNO started to bind to hM3D and neuronal activity gradually intensified. For the remainder of the continuous scanning session, the effect size peaked, predominantly displayed in red on the heatmap, indicating that neuronal activity remained elevated for an extended period post-CNO binding to hM3D, demonstrating significant differences between CNO-injected mice and the saline control group (**Figure 7B**).

For specific brain regions, such as the chemogenetically activated ipsilateral visual cortex (VC_R) (**Figure 7C**) and contralateral visual cortex (VC_L) (**Figure 7D**), effect sizes reached as high as +4.7, reflecting a very strong effect. The effect size statistics for other brain regions are shown in **Figure S1**.

### Temporal dynamics of chemogenetically activated ROIs

**Figure 8:**
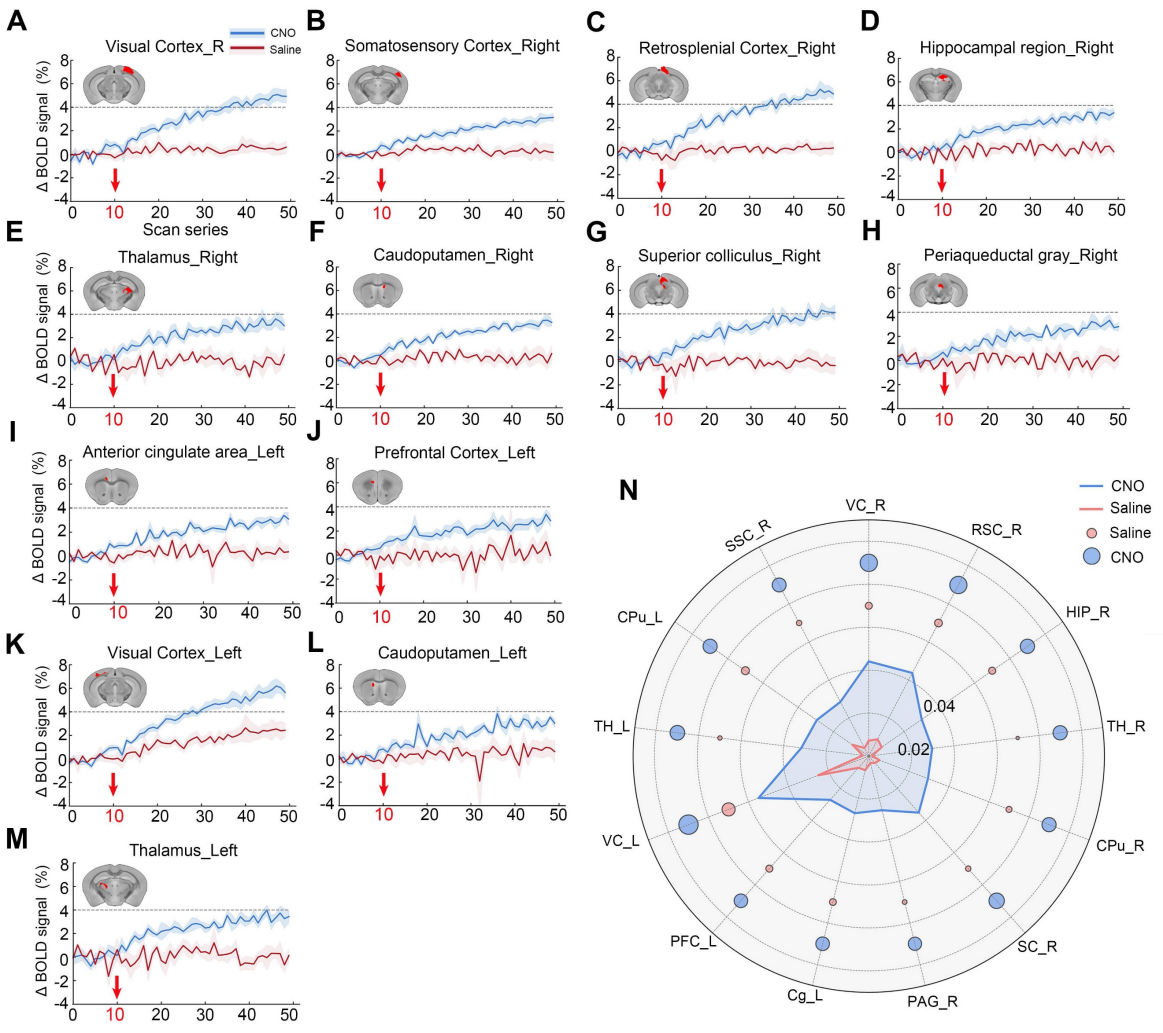
**Time series of BOLD signals in significantly chemogenetically activated brain regions.** (A) Average changes in BOLD signals within the virus-infected region (VC) of the V1 brain area following chemogenetic activation with CNO injection. Red arrows indicate the timing of CNO (blue line) or saline (red line) administration; (B-M) Significant changes in BOLD signals were observed in various brain regions, including the TH (B), RSC (C), right HIP (HIP_R, D), right TH (TH_R, E), right CPu (CPu_R, F), right SC (SC_R, G), right PAG (PAG_R, H), left Cg (Cg_L, I), left PFC (PFC_L, J), left VC (VC_L, K), left CPu (CPu_L, L), and left TH (TH_L, M); (N) The increase in BOLD signals in brain regions following chemogenetic activation. *Note*: repetition number = 50, one scan = 1 min 32 s.

To more clearly observe the temporal changes in neuronal activity induced by chemogenetic activation following CNO administration, the average time series of the activated regions was extracted (**Figure 8A-8M**). After CNO injection (at the 10th time point of scanning), the BOLD signal increased and remained elevated throughout the scanning session, meaning up to one-hour post-CNO injection (**Figure 8A-8M**). No significant response was observed in the saline-treated hM3D-expressing animals (Saline group), indicating the specificity of CNO-induced activation (**Figure 8A-8M**). In the right V1 region, the measured BOLD response reached a plateau approximately 4% above baseline after 15-20 minutes of injection (**Figure 8A**). Similarly, in other regions such as TH and SC, a positive BOLD response also approached a plateau of about 4% above baseline (**Figure 8B, 8E and 8M**). Quantification of BOLD signal increases in the activated brain regions showed that saline had minimal effects on BOLD signal elevation. The BOLD response in the VC and RSC regions is relatively stronger than that in the SSC, TH, and CPu regions (**Figure 8N**).

## Discussion

Deciphering the structural and functional networks of neural connectivity in living animals is crucial for understanding their roles in information processing and behavioral regulation. This study presents an integrated approach that combines tracing neural connections with analyzing the functional networks of associated circuits *in vivo* and in the whole brain. In this study, rAAV-PHP.eB-DIO encoding AQP1 was utilized to locally infect the brain or the whole brain through crossing the blood-brain barrier. Through the Cre-dependent expression system, diffusion-weighted MRI (DWI-MRI) was employed to achieve precise *in vivo* imaging of the specific neural connections for a target brain region. Additionally, Cre recombinase was used to mediate the expression of rAAV9-EF1α-DIO-hM3D (Gq), enabling chemogenetic activation of the neural circuits of a target region V1. The resulting functional networks were assessed using fMRI. This integrated strategy provides a powerful tool and valuable insights for decoding the relationship between brain structure and function networks, and offers a deeper understanding of the complex interplay between neuronal activity and brain connectivity.

### MRI reporter genes for biological imaging

Currently, various biological reporter genes based on different MRI contrast mechanisms have been developed for advanced imaging of biological tissues. These techniques enable imaging of reporter gene activity at depths of several centimeters, with spatial resolutions in the range of 100 μm and temporal resolutions as high as hundreds of milliseconds 36. Among these reporters, the MRI reporter gene ferritin enhances superparamagnetic properties and significantly alters solvent nuclear magnetic resonance relaxation rates by capturing endogenous iron ions within cells 37. Previous studies have utilized retrograde viral vectors expressing ferritin, such as AAVs, to visualize neural connectivity 25. However, the ferritin-mediated MRI contrast has a relatively long latency period, with signal enhancement being indirectly achieved through iron ion accumulation. This process is dependent on iron ion availability, and its sensitivity is comparatively lower than that of direct contrast agents, such as gadolinium-based agents.

Proteins from the SLCO superfamily, such as Oatp1a1, demonstrate the ability to translocate various imageable small molecules across membranes 38. By transporting the clinically approved hepatobiliary MRI contrast agent gadolinium-ethoxybenzyl-DTPA (Gd-EOB-DTPA), Oatp1a1 has been shown to produce rapid, intense, and reversible signal enhancement in T1-weighted MR images 39. Despite its remarkable image contrast capability, Oatp1a1 remains reliant on exogenous contrast agents, which may not effectively reach all tissues expressing the reporter gene.

Another artificial reporter gene, lysine-rich protein, generates MRI contrast *via* chemical exchange saturation transfer (CEST) 40, although it cannot be synthesized endogenously by the organism. Urea transporter UTB and aquaporins have been employed as reporter genes by enhancing transmembrane water exchange to facilitate MRI imaging. Detection of UTB requires specialized filter-exchange imaging (FEXI) techniques to measure the re-equilibration of magnetization between extracellular and intracellular water compartments after exchange 41, a process that involves a relatively complex signal acquisition. In contrast, aquaporin 1 (AQP1) generates magnetic resonance signals by increasing the diffusion rate of water within cells and can be detected using conventional ¹H MRI scanners and diffusion MRI pulse sequences 42. Recent studies have demonstrated that AQP1 is suitable for tracking tumor-specific gene expression 43, mapping neural connectivity 26, and constructing whole-brain atlases of astrocyte populations in mice 44. In this study, AQP1 was delivered across the blood-brain barrier *via* an AAV vector capable of crossing this barrier, enabling whole-brain delivery. Following Cre recombinase-mediated excision, AQP1 facilitated anterograde monosynaptic neural circuit tracing. The findings highlight AQP1's high sensitivity as an MRI reporter gene and its robust capacity for targeted visualization of specific brain regions.

### *In vivo* mapping structural network using gene integration with Cre/l*ox* system

The mammalian central nervous system (CNS) comprises a highly diverse array of neuronal types, which can be distinguished by their intrinsic gene expression profiles, differentially regulated throughout development. Integration of the Cre/*Lox* system could facilitate precise genetic manipulation and specific labeling of neural circuits. Targeted manipulation strategies typically incorporate classical genetic tools (such as the Cre*/Lox* and Flp/*FRT* systems), the use of cis-regulatory elements, and gene delivery *via* transgenic mouse models or viral vectors such as AAV 45. In the present study, AAV1-Cre was used in combination with AAV carrying AQP1 to enable *in vivo* visualization of monosynaptic connections downstream of the injection site in the V1 region, effectively crossing the blood-brain barrier. Furthermore, AAV1 has been shown to successfully transduce both excitatory and inhibitory cell types within GABAergic projection pathways, including projections from the striatum to the substantia nigra reticulata, as well as from the substantia nigra reticulata to the ventrolateral nucleus 46. Pathways involving pontine nuclei (PN) neurons projecting to the cerebellar granule layer have also been effectively mapped using these strategies 47. This method holds future potential for tracing anterograde monosynaptic neural connections of other cell types and visualizing these circuits through MRI.

Beyond the use of Cre-mediated viral tracing in C57BL/6 mice for labeling neural circuits, other Cre-driver transgenic mouse models, such as Vglut2-Cre and DAT-Cre, could be employed to dissect neural connections of different neuronal subtypes 48, 49. Additionally, activity-dependent transgenic mouse models such as FosTRAP2 could also be utilized to label neurons responsive to specific stimuli (e.g., water deprivation or fear responses) 50, 51. When combined with AQP1, which crosses the blood-brain barrier, this strategy could potentially facilitate the coupling of AQP1 with *c*Fos responses indicative of neuronal activity.

### Targeted activation effects of afferent and efferent projections of V1

This study utilized DREADD technology to active the neurons in V1, aiming to establish a causal link between localized genetically targeted chemogenetic activation and dynamic changes in the whole-brain neural networks. By comparing and analyzing the changes in monosynaptic neural connectivity networks marked by anterograde AQP1 labeling and functional networks, we inferred potential whole-brain network responses resulting from V1 activation.

Ten minutes after the injection of CNO, BOLD signals from the whole-brain fMRI showed significantly enhanced activity in multiple brain regions, including CPu, HIP, TH, SSC, VC, SC, LGN and PAG. The enhanced LGN activity may be partially attributed to its role in receiving substantial visual input from the retina and sending projections to V1, potentially contributing to the observed network activity changes 52. This may explain the significant enhancement of AQP1-EGFP-labeled DWI signals in the LGN region compared to other regions following CNO administration.

In addition, other regions exhibiting enhanced BOLD signals, such as the CPu, TH, and SC, are the downstream projection targets of V1. Literature reports suggest that subcortical structures such as the SC and PAG play crucial roles in visuomotor integration and autonomic responses. The activation of these regions may be mediated through both the retina-thalamus-visual cortex pathway and the tecto-cortical circuit 53, 54. Moreover, higher-order downstream networks, including PFC, Cg, and HIP, also exhibited significant responses to V1 activation. These regions within the higher-order downstream networks may be involved in more advanced cognitive functions and emotional regulation through complex network integration 55-58.

Overall, V1, through its complex afferent and efferent projections, establishes extensive functional network connections with multiple cortical and subcortical regions. This indicates the significant role of V1 in visual processing and suggests the potential applicability of this research approach to other neural circuitry studies. These findings lay an important foundation for further exploration of the functional mechanisms of V1-related networks and their role in behavioral regulation.

### Applications of AQP1 in disease diagnostics

Aquaporins (AQPs) regulate the diffusion of water molecules across cell membranes, providing a sensitive mechanism for modulating DWI signals. This property is crucial for high-resolution and dynamic imaging, particularly in applications related to *in vivo* detection 42. As an MRI reporter gene, AQP1 has demonstrated distinct advantages in *in vivo* detection of neural network or tumors. A lentiviral system based on the hTERT promoter and AQP1 gene (hTERT-AQP1) has been successfully applied for non-invasive* in vivo* visualization of xenografted subcutaneous malignant tumors, showing significant contrast enhancement when combined with DWI imaging. Furthermore, a Cre-*loxP*-controlled lentiviral vector expressing the oncogenes Harvey-Ras (H-Ras) and activated AKT was used to transduce as few as 60 GFAP+ cells into the subventricular zone or hippocampus of adult immunocompetent mice, leading to the induction of tumors resembling high-grade gliomas (WHO grade III-IV) 59. Therefore, the combination of AQP1 holds promise for early detection and long-term dynamic monitoring of the tumor, especially for gliomas induced by oncogene mutations.

## Conclusions

By combining DWI-MRI and chemogenetic-fMRI, this study successfully mapped the structural and functional networks of a target brain region V1. The integration of BOLD-fMRI with structural imaging allowed for the visualization of the impact of V1 activation on both local and extended neural networks, demonstrating the utility of this combined methodology. The results highlighted the role of V1 in visual information processing, particularly its complex network connections with multiple cortical and subcortical regions. This approach provides a promising pathway for investigating other neural circuits and offers an initial direction for future researches on the functional mechanisms underlying brain network formation and their role in behavioral regulation. Furthermore, deciphering the structural and functional connectivity of brain networks is also crucial for understanding the variations of brain function under physiological or pathological conditions.

## Supplementary Material

Supplementary figure and table.

## References

[1] Wei W, Deng L, Qiao C, Yin Y, Zhang Y, Li X (2023). Neural variability in three major psychiatric disorders. Mol Psychiatry.

[2] Tang H, Ma G, Zhang Y, Ye K, Guo L, Liu G (2023). A comprehensive survey of complex brain network representation. Meta Radiol.

[3] Mori S, Zhang J (2006). Principles of diffusion tensor imaging and its applications to basic neuroscience research. Neuron.

[4] Benes F, Berretta S (2001). GABAergic interneurons: Implications for understanding schizophrenia and bipolar disorder. Neuropsychopharmacol.

[5] Dauer W, Przedborski S (2003). Parkinson's disease: Mechanisms and models. Neuron.

[6] Zlokovic B (2011). Neurovascular pathways to neurodegeneration in Alzheimer's disease and other disorders. Nat Rev Neurosci.

[7] Nassi J, Cepko C, Born R, Beier K (2015). Neuroanatomy goes viral!. Front Neuroanat.

[8] Xu X, Holmes T, Luo M, Beier K, Horwitz G, Zhao F (2020). Viral Vectors for Neural Circuit Mapping and Recent Advances in Trans-synaptic Anterograde Tracers. Neuron.

[9] Liu Q, Wu Y, Wang H, Jia F, Xu F (2022). Viral Tools for Neural Circuit Tracing. Neurosci Bull.

[10] Srivastava A (2016). In vivo tissue-tropism of adeno-associated viral vectors. Curr Opin Virol.

[11] Salegio E, Samaranch L, Kells A, Mittermeyer G, San Sebastian W, Zhou S (2013). Axonal transport of adeno-associated viral vectors is serotype-dependent. Gene Ther.

[12] Wang D, Tai P, Gao G (2019). Adeno-associated virus vector as a platform for gene therapy delivery. Nat Rev Drug Discov.

[13] Zingg B, Chou X, Zhang Z, Mesik L, Liang F, Tao H (2017). AAV-Mediated Anterograde Transsynaptic Tagging: Mapping Corticocollicular Input-Defined Neural Pathways for Defense Behaviors. Neuron.

[14] Deverman B, Pravdo P, Simpson B, Kumar S, Chan K, Banerjee A (2016). Cre-dependent selection yields AAV variants for widespread gene transfer to the adult brain. Nat Biotechnol.

[15] Chan K, Jang M, Yoo B, Greenbaum A, Ravi N, Wu W (2017). Engineered AAVs for efficient noninvasive gene delivery to the central and peripheral nervous systems. Nat Neurosci.

[16] Yousaf T, Dervenoulas G, Politis M (2018). Advances in MRI Methodology. Int Rev Neurobiol.

[17] De Micco R, Russo A, Tessitore A (2018). Structural MRI in Idiopathic Parkinson's Disease. Int Rev Neurobiol.

[18] Oishi K, Mielke M, Albert M, Lyketsos C, Mori S (2011). DTI Analyses and Clinical Applications in Alzheimer's Disease. J Alzheimers Dis.

[19] Mukherjee A, Davis H, Ramesh P, Lu G, Shapiro M (2017). Biomolecular MRI reporters: Evolution of new mechanisms. Prog Nucl Magn Reson Spectrosc.

[20] Farhadi A, Sigmund F, Westmeyer G, Shapiro M (2021). Genetically encodable materials for non-invasive biological imaging. Nat Mater.

[21] Yun J, Baldini M, Chowdhury R, Mukherjee A (2022). Designing Protein-Based Probes for Sensing Biological Analytes with Magnetic Resonance Imaging. Anal Sens.

[22] Naumova A, Vande Velde G (2018). Genetically encoded iron-associated proteins as MRI reporters for molecular and cellular imaging. Wiley Interdiscip Rev Nanomed Nanobiotechnol.

[23] Song N, Zhang J, Zhai J, Hong J, Yuan C, Liang M (2021). Ferritin: A Multifunctional Nanoplatform for Biological Detection, Imaging Diagnosis, and Drug Delivery. Acc Chem Res.

[24] Zheng N, Su P, Liu Y, Wang H, Nie B, Fang X (2019). Detection of neural connections with *ex vivo* MRI using a ferritin-encoding trans-synaptic virus. NeuroImage.

[25] Cai A, Zheng N, Garth J, Wu Y, Nie B, Lin K (2021). Longitudinal neural connection detection using a ferritin-encoding adeno-associated virus vector and in vivo MRI method. Hum Brain Mapp.

[26] Zheng N, Li M, Wu Y, Kaewborisuth C, Li Z, Gui Z (2022). A novel technology for in vivo detection of cell type-specific neural connection with AQP1-encoding rAAV2-retro vector and metal-free MRI. NeuroImage.

[27] Logothetis N (2008). What we can do and what we cannot do with fMRI. Nature.

[28] Li B, Gong L, Wu R, Li A, Xu F (2014). Complex relationship between BOLD-fMRI and electrophysiological signals in different olfactory bulb layers. NeuroImage.

[29] Tu W, Cramer S, Zhang N (2024). Disparity in temporal and spatial relationships between resting-state electrophysiological and fMRI signals. Elife.

[30] Park H, Carmel J (2016). Selective Manipulation of Neural Circuits. Neurotherapeutics.

[31] Zerbi V, Floriou-Servou A, Markicevic M, Vermeiren Y, Sturman O, Privitera M (2019). Rapid Reconfiguration of the Functional Connectome after Chemogenetic *Locus Coeruleus* Activation. Neuron.

[32] Roelofs T, Verharen J, van Tilborg G, Boekhoudt L, van der Toorn A, de Jong J (2017). A novel approach to map induced activation of neuronal networks using chemogenetics and functional neuroimaging in rats: A proof-of-concept study on the mesocorticolimbic system. NeuroImage.

[33] Zheng N, Gui Z, Liu X, Wu Y, Wang H, Cai A (2023). Investigations of brain-wide functional and structural networks of dopaminergic and CamKIIα-positive neurons in VTA with DREADD-fMRI and neurotropic virus tracing technologies. J Transl Med.

[34] Tu W, Ma Z, Ma Y, Dopfel D, Zhang N (2021). Suppressing Anterior Cingulate Cortex Modulates Default Mode Network and Behavior in Awake Rats. Cereb Cortex.

[35] Tu W, Ma Z, Zhang N (2021). Brain network reorganization after targeted attack at a hub region. Neuroimage.

[36] Brindle K (2022). Gene reporters for magnetic resonance imaging. Trends Genet.

[37] Genove G, DeMarco U, Xu H, Goins W, Ahrens E (2005). A new transgene reporter for *in vivo* magnetic resonance imaging. Nat Med.

[38] Leonhardt M, Keiser M, Oswald S, Kuhn J, Jia J, Grube M (2010). Hepatic Uptake of the Magnetic Resonance Imaging Contrast Agent Gd-EOB-DTPA: Role of Human Organic Anion Transporters. Drug Metab Dispos.

[39] Patrick P, Hammersley J, Loizou L, Kettunen M, Rodrigues T, Hu D (2014). Dual-modality gene reporter for in vivo imaging. Proc Natl Acad Sci U S A.

[40] Gilad A, McMahon M, Walczak P, Winnard P, Raman V, van Laarhoven H (2007). Artificial reporter gene providing MRI contrast based on proton exchange. Nat Biotechnol.

[41] Schilling F, Ros S, Hu D, D'Santos P, McGuire S, Mair R (2017). MRI measurements of reporter-mediated increases in transmembrane water exchange enable detection of a gene reporter. Nat Biotechnol.

[42] Mukherjee A, Wu D, Davis HC, Shapiro MG (2016). Non-invasive imaging using reporter genes altering cellular water permeability. Nat Commun.

[43] Zhang L, Gong M, Lei S, Cui C, Liu Y, Xiao S (2022). Targeting visualization of malignant tumor based on the alteration of DWI signal generated by hTERT promoter-driven AQP1 overexpression. Eur J Nucl Med Mol Imaging.

[44] Li M, Liu Z, Wu Y, Zheng N, Liu X, Cai A (2024). In vivo imaging of astrocytes in the whole brain with engineered AAVs and diffusion-weighted magnetic resonance imaging. Mol Psychiatry.

[45] Haery L, Deverman B, Matho K, Cetin A, Woodard K, Cepko C (2019). Adeno-Associated Virus Technologies and Methods for Targeted Neuronal Manipulation. Front Neuroanat.

[46] Zingg B, Peng B, Huang J, Tao H, Zhang L (2020). Synaptic Specificity and Application of Anterograde Transsynaptic AAV for Probing Neural Circuitry. J Neurosci.

[47] Kanichay R, Silver R (2008). Synaptic and cellular properties of the feedforward inhibitory circuit within the input layer of the cerebellar cortex. J Neurosci.

[48] Chefer V, Bäckman C, Gigante E, Shippenberg T (2013). Kappa Opioid Receptors on Dopaminergic Neurons Are Necessary for Kappa-Mediated Place Aversion. Neuropsychopharmacol.

[49] Borgius L, Restrepo C, Leao R, Saleh N, Kiehn O (2010). A transgenic mouse line for molecular genetic analysis of excitatory glutamatergic neurons. Mol Cell Neurosci.

[50] DeNardo L, Liu C, Allen W, Adams E, Friedmann D, Fu L (2019). Temporal evolution of cortical ensembles promoting remote memory retrieval. Nat Neurosci.

[51] Allen W, DeNardo L, Chen M, Liu C, Loh K, Fenno L (2017). Thirst-associated preoptic neurons encode an aversive motivational drive. Science.

[52] Li Z, Peng B, Huang J, Zhang Y, Seo M, Fang Q (2023). Enhancement and contextual modulation of visuospatial processing by thalamocollicular projections from ventral lateral geniculate nucleus. Nat Commun.

[53] Beltramo R, Scanziani M (2019). A collicular visual cortex: Neocortical space for an ancient midbrain visual structure. Science.

[54] Seabrook T, Burbridge T, Crair M, Huberman A (2017). Architecture, Function, and Assembly of the Mouse Visual System. Annu Rev Neurosci.

[55] Cang J, Savier E, Barchini J, Liu X (2018). Visual Function, Organization, and Development of the Mouse Superior Colliculus. Annu Rev Vis Sci.

[56] Alexander A, Nitz D (2015). Retrosplenial cortex maps the conjunction of internal and external spaces. Nat Neurosci.

[57] Sieben K, Röder B, Hanganu-Opatz I (2013). Oscillatory Entrainment of Primary Somatosensory Cortex Encodes Visual Control of Tactile Processing. J Neurosci.

[58] Murray E, Bussey T, Saksida L (2007). Visual perception and memory: A new view of medial temporal lobe function in primates and rodents. Annu Rev Neurosci.

[59] Marumoto T, Tashiro A, Friedmann-Morvinski D, Scadeng M, Soda Y, Gage F (2009). Development of a novel mouse glioma model using lentiviral vectors. Nat Med.

