# Supplementary material for: Investigating the relationships of structural and functional neural networks of primary visual cortex with engineered AAVs and chemogenetic-fMRI techniques
Source: Theranostics. 2025 Mar 3;15(9):3821–36. doi: 10.7150/thno.109625 (PMC11980648; doi:10.7150/thno.109625)
Supplement: Supplementary file 1 — Supplementary figure and table. [file thnov15p3821s1.pdf]

Supplementary Materials

Table S1. List of 48 regions of interest (ROIs) yielding significant findings from chemogenetic-fMRI analysis.

NO	Region	L/R	NO	Region	L/R
1	Anterior cingulate area	Right	25	Periaqueductal gray	Right
2	Orbital area	Right	26	raphe nuclei	Right
3	Prelimbic area	Right	27	Anterior cingulate area	Left
4	Infralimbic area	Right	28	Orbital area	Left
5	Primary motor area	Right	29	Prelimbic area	Left
6	Secondary motor area	Right	30	Infralimbic area	Left
7	Somatosensory area	Right	31	Secondary motor area	Left
8	Visceral area	Right	32	Somatosensory area	Left
9	Auditory area	Right	33	Auditory area	Left
10	Visual Cortex	Right	34	Visual Cortex	Left
11	Retrosplenial system	Right	35	Retrosplenial system	Left
12	Agranular insular area	Right	36	Temporal association areas	Left
13	Perirhinal area	Right	37	Piriform area	Left
14	Piriform area	Right	38	Hippocampal region	Left
15	Hippocampal region	Right	39	Thalamus	Left
16	Thalamus	Right	40	Retrohippocampal region	Left
17	Retrohippocampal region	Right	41	Cortical amygdalar area	Left
18	Cortical amygdalar area	Right	42	Amygdala	Left
19	Amygdala	Right	43	Caudoputamen	Left
20	Caudoputamen	Right	44	Septum	Left
21	Septum	Right	45	Superior colliculus	Left
22	Pallidum	Right	46	Inferior colliculus	Left
23	Superior colliculus	Right	47	Periaqueductal gray	Left
24	Inferior colliculus	Right	48	raphe nuclei	Left

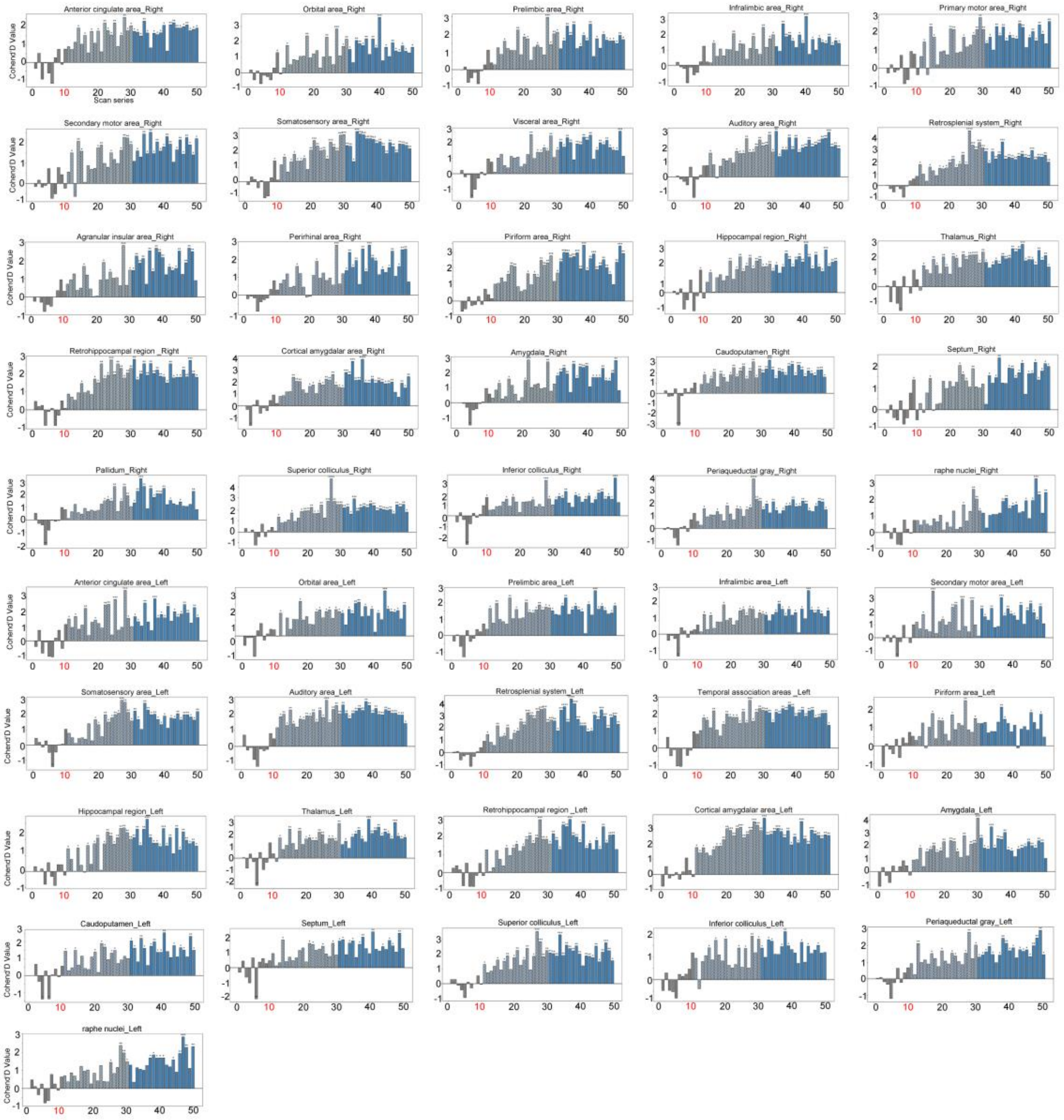

Figure S1. Statistical analysis of the effective 46 regions of interest (ROIs).

*N=6, * p < 0.05; ** p < 0.01; *** p < 0.001; **** p < 0.0001*
